# Midwives’ compliance with post-exposure prophylaxis guidelines in Tshwane District, South Africa

**DOI:** 10.4102/curationis.v47i1.2548

**Published:** 2024-09-11

**Authors:** Mosehle S. Matlala, Thanyani G. Lumadi

**Affiliations:** 1 Department of Health Studies, College of Human Sciences, University of South Africa, Tshwane, South Africa

**Keywords:** assessment, attitudes, guidelines, knowledge, midwifery practitioners, perceptions, post-exposure prophylaxis

## Abstract

**Background:**

Human immunodeficiency virus (HIV) remains a major public health concern. Midwifery practitioners as frontline healthcare workers (HCWs) remain susceptible to occupational exposure to infections while performing their routine duties. It is estimated that 90% of occupational exposures occur because of a lack of awareness and training regarding prevention and measures to be taken in case of accidental exposure.

**Objectives:**

The study aimed to assess the knowledge, attitudes and compliance of midwifery practitioners regarding post-exposure prophylaxis (PEP) guidelines.

**Method:**

Concurrent mixed-methods research approach with qualitative nested in quantitative design was followed. A random simple sampling technique was used to collect quantitative data from 71 midwifery practitioners. Simultaneously, a purposive non-probability sampling technique was used for the qualitative approach with two occupational health and safety (OHS) practitioners and 13 midwifery practitioners. Data were collected through questionnaires and semi-structured interviews. Quantitative data were analysed with SPSS version 24 and presented in tables and figures, and thematic analysis was employed for the qualitative strand.

**Results:**

The midwifery practitioners have good knowledge about PEP for HIV. However, the study revealed the underreporting of accidental exposures to blood and body fluids (BBFs) and the underutilisation of available PEP services.

**Conclusion:**

Maternity units are high-risk clinical environments. Underreporting of incidents of exposure remains prevalent among midwifery practitioners.

**Contribution:**

The findings will inform policy development structures and hospital management regarding knowledge and implementation gaps related to PEP guidelines in the specific hospitals. Strategies to improve compliance with PEP among midwifery practitioners were developed as a derivative from study findings.

## Introduction

About 36 million people are living with human immunodeficiency virus (HIV) worldwide, and South Saharan Africa alone accounts for over 25 million (70%), making it the region with the biggest disease burden (Babanawo et al. 2018:2). HIV and acquired immunodeficiency syndrome (AIDS) remains a major public health problem costing the lives of many people, including healthcare workers (HCWs). Healthcare workers are at high risk of being infected with various diseases transmitted by blood and body fluids (BBFs) because of frequent exposure to biological materials and patient’s body fluids (Yasin et al. 2019:2). South Africa (SA) has the highest number of people living with HIV in the world, estimated at 7.7 million (Tekalign et al. 2022:812). Occupational exposure to HIV can be prevented through compliance with the precautionary measures related to clinical practice. Compliance is the extent to which certain healthcare practices are implemented following known recommendations (Ayele et al. 2022:2).

Occupational risks associated with exposure to BBFs also affect the quality of care delivered, as well as the safety and well-being of HCWs (Tekalign et al. 2022:812). The risk of exposure to HIV while performing routine duties in the clinical environment is inherently high in midwifery practice (Cao et al. 2020:228). The most effective safety measures to prevent contamination with BBFs include precautionary and safety measures, safe-needle procedures, barriers and other preventive strategies (Shakeel et al. 2022:2).

Globally, occupational exposure to blood-borne pathogens because of contact with human BBFs has become a serious health concern for HCWs (Mengistu et al. 2022:1). Moreover, about three million HCWs are exposed to blood-borne pathogens each year, of which 170 000 are exposed to HIV infections (Mengistu et al. 2022:2). HCWs are more susceptible to contracting infections because of the nature of the critical care environment and, frequently, close contact with patients and invasive procedures exposes them to body fluids and infectious microorganisms (Mulat et al. 2022:1).

An increasing number of midwifery practitioners is affected by occupational exposures (Shitu, Adugna & Abebe 2021:2). Midwives are more frequently exposed to patients’ BBFs, secretions, amniotic fluid and sharps than general ward nurses, and their risk of blood-borne occupational exposure is greater (Li et al. 2023:214). Standard precautions are designed to protect HCWs and patients from exposure to BBFs (Ayele et al. 2022:2). Therefore, it is crucial for HCWs to adhere to standard precautions. In the unfortunate event that an individual is exposed to BBFs, the guidelines prescribe that HIV post-exposure prophylaxis (PEP) should be offered and initiated as early as possible, preferably within 72 h (WHO 2021:88).

South Africa has long been contributing to clinical practice guidelines development with key players, including the Department of Health and other stakeholders, producing guidance for their respective constituents (Kredo et al. 2017:2). Nurses in SA deviate from clinical guidelines in a variety of ways, including a lack of knowledge and training (Makhado, Davhana-Maselesele & Farley 2018:3). Globally, approaches for adapting guidelines are not clear (McCaul et al. 2020:193). Considering the inherent risk of exposure to BBFs in midwifery practice, there is insufficient evidence of research conducted regarding the experiences and responses to occupational exposures. Literature shows that most studies conducted in Southern Africa about occupational exposure to HIV focused mainly on HCWs collectively. Therefore, it interested the researchers to delve into the space of midwifery practitioners.

Dankwa and Nakata’s Compliance Assessment Framework (2018) was adopted in this study to analyse the behaviour and attitudes of midwifery practitioners towards the implementation of PEP guidelines. The objective of the study was to assess the attitudes, knowledge and perceptions of midwifery practitioners regarding PEP guidelines, with the aim of developing strategies to improve compliance with PEP.

## Research methods and design

### Study design

The study used mixed-methods research, which involved collecting, analysing and in some way integrating both quantitative and qualitative data (Leavy 2017:9). The mixed-methods approach links qualitative and quantitative designs to create a more holistic understanding (Fetters & Molina-Azorinas 2017:293). The approach in this study took a concurrent nested design, where the main research method was used with another to answer different research questions or focus more on a minor group (*Barnes* in Maarouf 2019:4). Qualitative nested in quantitative designs involve using a quantitative method as the primary method while nesting a qualitative component in the design (Leavy 2017:263).

The study adopted the non-experimental quantitative research with a cross-sectional descriptive design and a descriptive phenomenological design for the qualitative strand.

### Setting

The study was conducted in three hospitals in the Tshwane District of the Gauteng province SA. The selected hospitals’ categories, as gazetted, were the district, regional and provincial tertiary hospitals. The categories differed in their scope of the package of service in terms of the level of speciality healthcare rendered. The common factor among the three hospitals is that they all offered, among other services, maternity services, namely antenatal, labour and postnatal wards, with regional and provincial hospitals offering neonatal intensive care units as well.

### Study population and sampling strategy

The study population included midwifery and occupational health and safety (OHS) practitioners working at the three selected public hospitals in Tshwane District. Because of the nature of the nested design, a cohort of OHS practitioners from each hospital was included in the study to corroborate the findings from the midwifery practitioners. The total population in the study comprised 133 registered midwifery practitioners on the sampling frame; using the online calculator for sample size determination with a confidence level of 95% and confidence interval of 5, the sample size needed was 99.

#### Sampling for quantitative strand

A simple random probability sampling technique was used in the study. From the 99 questionnaires sent to midwifery practitioners, only 71 midwifery practitioners responded to the questionnaires, which is an approximately 72% response rate. The target sample was midwifery practitioners working in the maternity units, that is, labour, and postnatal wards.

#### Sampling for qualitative strand

A purposive non-probability sampling technique was used to sample the 3 OHS practitioners and 13 midwifery practitioners, 6 from provincial tertiary, 5 from the district and 2 from the regional hospitals to participate in interviews. The researcher had planned to interview 3 OHS practitioners, 1 from each hospital but could not get consent from 1. Only 2 OHS practitioners, one from the district and one from the regional hospital were ultimately interviewed. Among the midwifery practitioners that participated in the semi-structured interviews, only 2 did not take part in quantitative data collection.

### Data collection

Following permission to conduct the study at the selected sites, data collection was approached in a parallel fashion. The pretesting of the questionnaire and the interview guide was conducted to validate whether the content of the data-collection tools was relevant. Pretesting is used by researchers to evaluate and improve the data-collection instruments (Campoamor et al. 2024:110). In this study, the researcher tested the questionnaire with five midwifery practitioners working in the neonatal intensive care unit of one of the hospitals selected for the study. Quantitative and qualitative data-collection processes were conducted concurrently and independently. Questionnaires were used for the quantitative data collection, while semi-structured interviews were conducted for qualitative data collection. Completed questionnaires collected from district, regional and tertiary hospitals were 15, 24 and 32, respectively. A total of 71 questionnaires were completed.

The questionnaire, as guided by literature and PEP guidelines, covered four sections, namely, the participants’ socio-demographic data, knowledge about PEP, attitudes towards PEP and compliance with PEP guidelines. Apart from the socio-demographic data, included in the interview guide, were questions related to the common types of occupational exposures in maternity units, immediate procedures and management of side effects of antiretroviral drugs.

Semi-structured interviews were conducted telephonically with nine midwifery practitioners and two OHS practitioners, while face-to-face interviews with the other four midwifery practitioners were conducted in the duty room. All sessions were recorded, and notes were taken in the process. Privacy was maintained throughout the interviews, as all staff on duty in the same shift were made aware of the interviews in progress to avoid interruptions.

### Data analysis

#### Quantitative strand

A Microsoft Excel sheet was used for data entry. Data cleaning and analysis were performed using the Statistical Package for Social Sciences (SPSS) version 24. Frequencies and charts were used to summarise responses from all sections of the questionnaire.

#### Qualitative strand

The researcher followed a six-step phase approach in conducting a thematic analysis of collected data from the semi-structured interviews. The six phases, as described in Nowell et al. (2017:4), are: (1) familiarising with data, (2) generating initial codes, (3) searching for themes, (4) reviewing themes, (5) defining and naming themes and finally, (6) producing report. The researcher followed Schutz’s postulate of subjective interpretation in line with preserving the participant’s subjective point of view and acknowledging the context within which the phenomenon is studied (Fereday & Muir-Cochrane 2006:82).

The interview responses from participants were quoted verbatim under the three emerged themes, namely, perceptions on PEP guidelines, reporting incidents and seeking treatment and support. After that, categories were created, and the themes were applied to the rest of the data (Kibiswa 2019:2060). There was no need to go back to the participants for further clarification. Integration of quantitative and qualitative results was achieved using a convergent design and a merging approach.

### Ethical considerations

Ethical clearance was received from the University of South Africa’s College of Human Sciences Research Ethics Review Committee (reference no.: 40667235_CREC_CHS_2021) and the Tshwane Research Committee (reference no.: GP_202109_048). The National Health Research Database reference number was allocated (reference no.: GP_202109_048) following the uploading of the protocol onto the database. Approval to conduct research at the district and regional hospitals was granted by the Tshwane Ethics Committee (project no.: 68/2021). Permission to conduct research at the tertiary institution was granted with reference number KPTH/MARCH 2022. The questionnaires were coded with alphanumeric codes to secure the anonymity of participants. Pseudonyms were used for the semi-structured interviews. Formal written consent was obtained from each respondent before engaging with the questionnaire and participating in the interviews. Participants were encouraged to exercise their autonomy regarding voluntary participation and withdrawal from the study at any time they wished to. The philosophy underpinning autonomy is that all persons have intrinsic and unconditional worth; therefore, they should have the power to make rational decisions and moral choices (Varkey 2021:19). This study bore no risk for harm to participants, rather the researchers aimed to use the findings to develop strategies to enhance compliance with PEP, which were anticipated to benefit midwifery and OHS practitioners.

#### Trustworthiness

In establishing trustworthiness, Lincoln and Guba created stringent criteria known as credibility, dependability, confirmability and transferability (Forero et al. 2018:2). In ensuring integrity and reliability, the researcher was committed to the ethical principles from preparation, organisation and reporting of results. The components of trustworthiness are described briefly further in the text.

#### Credibility

Using a qualitative-quantitative approach, triangulation of the data was performed, and therefore the credibility of the study was achieved.

#### Dependability

This study was developed from the early stages through a systematic search of the existing literature about occupational exposures to BBFs and the management of such incidents through the utilisation of PEP services among HCWs. All completed data-collection tools were kept safe and separately according to the study sites to keep an easy audit trail.

#### Confirmability

Verbatim accounts from the participants were recorded, and notes were taken at the same time. Codes and themes were derived during data analysis for the purpose of the audit trail. Corroboration of findings from the quantitative strand was performed through the nested approach undertaken in the study.

#### Transferability

The sampling method used for this study started with a simple random sampling technique for the quantitative part of the study, where participants were selected randomly from the duty roster of the three selected sites. Because of the nature of the mixed-methods research with a nested approach, a purposive non-probability sampling technique was used to sample participants required for the semi-structured interviews.

#### Reliability and validity of data-collection process

This study’s design, sampling, development of the study instruments, data collection and analysis phases all included measures that enhanced the study’s validity and reliability.

**External validity:** External validity is the degree to which the results of the study can be generalised to settings or samples other than the one studied (Polit & Beck 2017:728). To maximise the response rate, the researcher visited the research sites and engaged participants in an explanation of the study’s objectives. The researcher anticipated that the results in this study might not be generalisable to the whole population of the involved cohort of cadres as the study was limited to only three hospitals in the Gauteng province of SA.

**Internal validity:** Internal validity is the degree to which an inference can be made that an experimental intervention (independent variable), rather than confounding factors, caused the observed effects on the outcome (Polit & Beck 2017:731). The study design and implementation phases of this research, which included the synthesis of the theoretical framework, the identification of pertinent study variables and the development of the data-collection tool, the preparation for data-collection pretesting and data analysis, all addressed internal validity.

## Results

### Quantitative strand

#### Part 1: Socio-demographic characteristics

Socio-demographic factors are regarded, in some instances, as the predictors of adherence; hence, a few characteristics are included in this study (Turk et al. 2023:7772). All respondents worked in the maternity units at their respective hospitals, with 64.8% (*n* = 46) in the labour ward and 35.2% (*n* = 25) in the postnatal ward. Out of the 71 respondents, only 5.6% (*n* = 4) were males, and the rest 94.3% (*n* = 67) were females. Their age ranged from 23 years to 65 years old. About 32.3% (*n* = 23) held a basic midwifery qualification, while 67.6% (*n* = 48) had a post-basic midwifery qualification, also known as advanced midwifery. Table 1 depicts the characteristics of respondents.

**TABLE 1:** Socio-demographic characteristics of study participants: Quantitative and qualitative strands.

Variable	Quantitative strand (*N* = 71)		Qualitative strand (*N* = 15)
	*n*	%	*n*
**Age (years)**			
20–29	17	23.9	3
30–39	18	25.3	2
40–49	12	16.9	6
> 50	24	33.8	4
**Gender**			
Female	67	94.3	15
Male	4	5.6	0
**Marital status**			
Married	42	59.1	9
Single	27	38.0	6
Divorced	2	2.8	0
**Years of experience in maternity**			
2–5	23	32.3	3
6–10	19	26.7	3
> 10	29	40.8	9
**Area of allocation**			
Labour ward	46	64.7	7
Postnatal ward	25	35.2	2
Occupational health and safety	0	0.0	2
**Professional status**			
Basic midwifery	48	67.6	6
Post-basic midwifery	23	32.3	3
Occupational health and safety qualification	0	0.0	2

*Source:* Matlala, M.S., 2023, ‘Strategies to improve compliance to post exposure prophylaxis guidelines for midwifery practitioners at specific hospitals in Gauteng province, South Africa’, PhD thesis, Dept. of Health Studies, University of South Africa.

#### Part 2: Knowledge about PEP

All 71 respondents in the study indicated having heard about the HIV PEP guidelines. The objective of this question was to derive a base of knowledge from the respondents and to determine whether they had any information about PEP. A total of 73% (*n* = 52) respondents were aware of the availability of PEP guidelines, while 10% (*n* = 7) and 17% (*n* = 12) did not know or were unsure of the availability of guidelines, respectively. An average of 72% (51) of respondents believed that PEP has the potential to prolong life, whereas 13% (*n* = 9) did not perceive PEP as having the potential to prolong life, with 16% (*n* = 11) being uncertain of its potential.

These responses are evidence that some of the respondents will not adhere to the PEP guidelines. A proportion of 75% (*n* = 53) of midwifery practitioners regarded PEP as effective in preventing HIV. Three percent (*n* = 2) did not regard PEP as effective, while 23% (*n* = 16) were not sure. Although most respondents regarded PEP as an effective measure to prevent HIV, there was still a notable gap in knowledge about the purpose of PEP. This is congruent with findings among HCWs in Ethiopia, where the majority had moderate and poor knowledge of PEP (Shamil, Legese & Tadiwos 2021:50).

##### Direct exposures to blood and body fluids in maternity units

**FIGURE 1:**
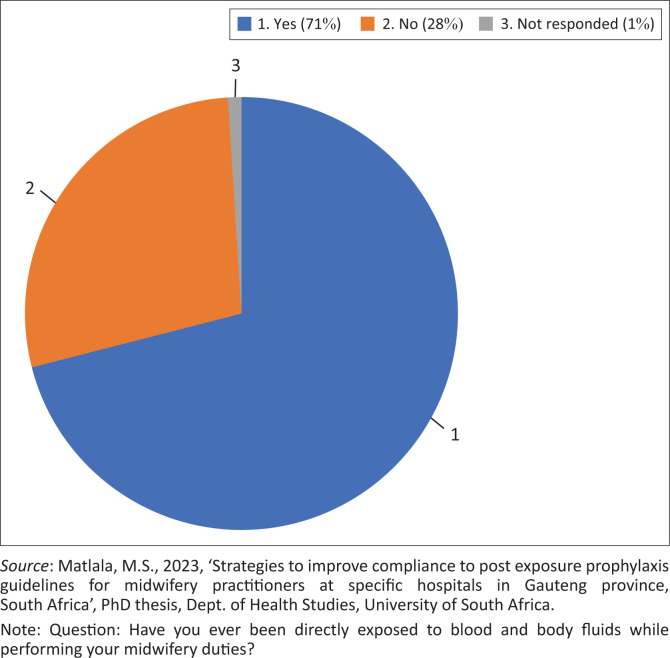
Direct exposures to blood and body fluids.

About 71% (*n* = 50) of respondents indicated that they had been directly exposed to BBFs at least once in their line of duty (Figure 1). A minimum of 28% (*n* = 20) responded that they had never been exposed to patients’ BBFs. One respondent did not answer this question. Of those directly exposed, 68% (*n* = 34) performed first aid and 32% (*n* = 16) did not. These numbers show that most midwifery practitioners are in danger of accidental exposure to BBFs while performing their midwifery duties.

##### Awareness of reporting procedures

Although 86% (*n* = 61) of the respondents were aware of the procedure for reporting the incidents of exposure as communicated to all personnel, at least 9% (*n* = 6) were not aware of the reporting procedures, and 6% (*n* = 4) were not sure if the procedures had ever been communicated to them. In this cohort of the 70% (*n* = 50) of respondents who were exposed to BBFs, at least 70% (*n* = 35) reported the incidents, while 21% (*n* = 15) did not report incidents. These reported poor trends and patterns of the use of the PEP regimen portray danger for the control and prevention of HIV (Adebimpe 2018:107).

##### Maternity units are perceived as high risk for blood and body fluids exposure

Midwifery practitioners view their work environment as high risk, with 92% (*n* = 65) who indicated in their response that exposure to BBFs makes their environment high risk. At least 6% (*n* = 4) did not view the environment as high risk, while 3% (*n* = 2) were not sure. The participants asserted that accidental exposures among colleagues in the maternity units occur more frequently, during suturing perineal tears and episiotomies. Most needle-stick injuries were reported to occur frequently while performing wound suturing (Aigbodion, Motara & Laher 2019:4).

##### Training on implementation of guidelines

**FIGURE 2:**
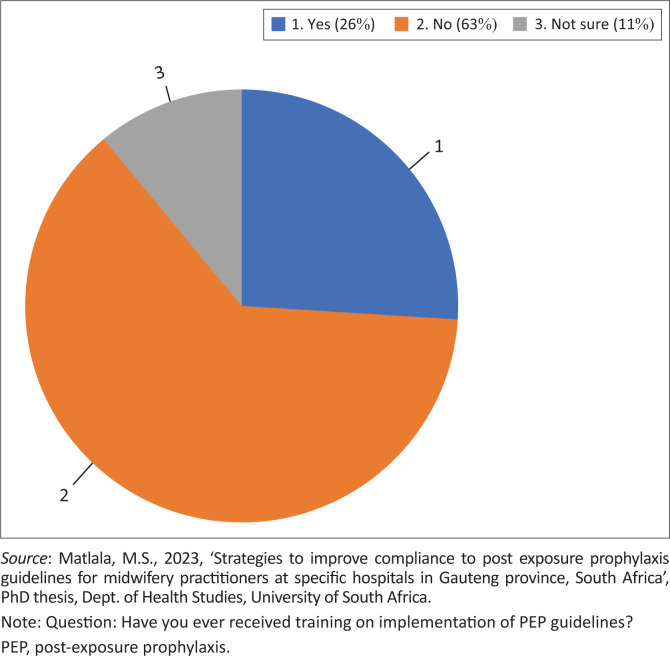
Proportions of trained versus untrained respondents.

Figure 2 shows the proportion of trained and untrained individuals on the implementation of PEP guidelines. A total of 19 participants responded to the question of whether training was provided or not. Training on the implementation of PEP guidelines seemed minimal, as evidenced by the response from 63% (*n* = 12) of respondents who answered ‘no’ to the question and 26% (*n* = 5) on training who responded that training was provided. Pre-service formal education programmes should incorporate HIV PEP guidelines and their compliance (Makhado et al. 2022:6).

More than three quarter, 71% (*n* = 50) were directly exposed to BBFs. At least 76% (*n* = 38) of the respondents acknowledged that counselling was offered on risks, benefits, side effects and the importance of adherence before initiation of the PEP regimen (Figure 2). Only 10% (*n* = 5) indicated that counselling was not provided while 14% (*n* = 7) did not respond. Accordingly, in the event of occupational exposure, HCWs who wish to take PEP should have adequate information and counselling that would assist them to benefit from PEP and prevent HIV transmission (Adebimpe 2018:107). Among the respondents directly exposed to BBFs, 68% (*n* = 34) received PEP from their place of work, while 24 % (*n* = 12) did not seek onsite PEP services. About 8% (*n* = 4) of participants did not respond to the question. As in Rasweswe and Peu (2020:4), some nurses chose to seek PEP services outside of their hospital, but others were able to access them onsite.

##### Completion of prescribed post-exposure prophylaxis course

About 72% (*n* = 36) of respondents indicated that they finished the course of PEP as prescribed, while 8% (*n* = 6) mentioned that they could not complete the course. About 16% (*n* = 8) did not respond to the question, which could indicate that they never had to be on PEP, or some had sought the services elsewhere.

#### Part 3: Attitudes towards post-exposure prophylaxis

Generally, most respondents showed a positive attitude towards PEP. Uncertainty exists over whether the positive outlook stems from a thorough comprehension of PEP or from using PEP to reduce the potential risk of infection.

##### Perceptions on the likelihood of post-exposure prophylaxis to reduce chances of human immunodeficiency virus infection

An average of 47% (*n* = 33) believed that PEP reduces the likelihood of HIV. About 13% (*n* = 9) disagreed, while 39% (*n* = 28) of those who responded were not sure if PEP could reduce the chances of HIV infection. Only one did not respond to the statement. It is, however, a concern that such a high number of respondents had a negative perception of PEP.

##### The effectiveness of personal protective equipment in preventing human immunodeficiency virus

The respondents were assessed to establish their perception of and attitudes towards using personal protective equipment (PPE) while performing duties that expose them to patients’ BBFs. A proportion of 86% (*n* = 61) agreed that the use of PPE is an effective measure to prevent the likelihood of HIV infection. A minimum of 3% (*n* = 2) did not agree and 11% (*n* = 8) responded that they were not sure. Personal protective equipment is specialised clothing worn by HCWs for protection against infection and provides a physical barrier when they are in contact with BBFs or discharges, non-intact skin or mucous membranes, soiled items and contaminated surfaces or equipment that might be infectious (Madziatera et al. 2020:124).

#### Part 4: Compliance with post-exposure prophylaxis

This marked the last portion of the questionnaire. The questions were aimed at establishing the behavioural intention of compliance with PEP among the respondents. The format of this part was also a Likert scale where participants had to respond with either agree or disagree.

##### Intentions to report and follow guidelines

Responding to the statement on reporting and following PEP guidelines in case of exposure to BBFs, 94% (*n* = 67) agreed, while 6% (*n* = 4) disagreed. A significant proportion of respondents seemed to be willing to report and follow the guidelines as outlined, albeit the study revealed that some of those who were exposed to the BBFs did not report the incidents. This means that individuals were aware of procedures to follow but consciously decided not to comply for various reasons. A higher percentage of midwifery practitioners are non-compliant with HIV PEP guidelines (Tsega et al. 2023:7).

##### Importance of follow-up testing for human immunodeficiency virus

After completion of the regimen, 97% (*n* = 69) agreed that it is important to have a follow-up HIV test to verify that the individual is not infected, and that PEP was effective. In comparison, 3% (*n* = 2) did not agree with the statement. Most of the respondents demonstrated a positive attitude towards a follow-up test after completion of prophylaxis. In certain cases, those who did not think a follow-up test was necessary can test elsewhere, confirm their negative status and choose to discontinue therapy, as was found in Makhado et al. (2022:4).

### Qualitative strand

#### Socio-demographic characteristics of participants: Qualitative strand

All participants, as shown in Table 1 and Table 2, were permanently employed at their respective selected study sites. Those who participated in semi-structured interviews ranged from 25 years to 55 years old and were female. The post-basic qualification in midwifery was held by older and longer service bearers in the field of midwifery. The OHS practitioners had a basic qualification in nursing and midwifery and an additional qualification in OHS.

**TABLE 2:** Semi-structured interview participants identifiers.

Participant code	Age (years)	Professional role	Hospital code
P1	49	M	H1
P2	33	M	H2
P3	33	M	H3
P4	50	O	H1
P5	55	O	H2
P6	31	M	H3
P7	25	M	H1
P8	41	M	H2
P9	47	M	H1
P10	50	M	H3
P11	44	M	H3
P12	46	M	H3
P13	36	M	H2
P14	54	M	H2
P15	34	M	H2

Note: Pseudonyms were created in relation to the participants’ codes, professional roles and sites (e.g. P1MH1).

M, midwifery practitioner; O, occupational health and safety practitioner.

#### Data analysis and interpretation

**FIGURE 3:**
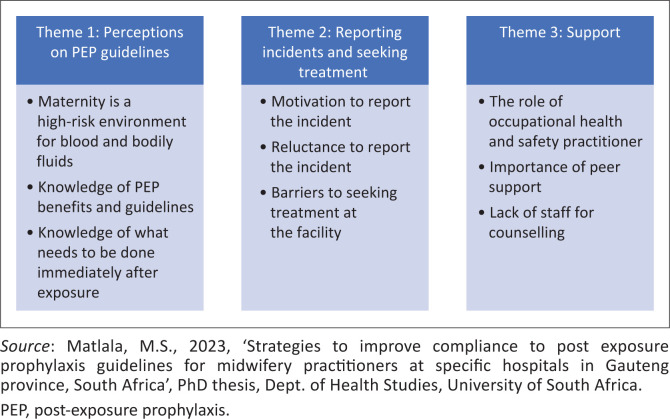
Themes and sub-themes.

A summary of themes and sub-themes is shown in Figure 3. Thematic analysis was conducted with the aim to systematically organise, analyse and search for themes that can capture the narratives available in the account of qualitative data sets (Dawadi 2020:62). Three main themes with eleven sub-themes emerged and are discussed further in the text with the verbatim responses from the participants.

#### Theme 1: Perceptions about post-exposure prophylaxis guidelines

The participants had varied opinions regarding the HIV PEP guidelines. Their shared experience mainly pointed to the risks and knowledge of PEP and its implementation.

##### Maternity is a high-risk environment for blood and body fluids

The midwifery practitioners asserted that the frequency of accidental exposures occurs among colleagues in the maternity units, mostly during suturing of perineal tears and episiotomies. This is consistent with the study in Ghana, where the majority of HCWs were reported to have been at risk of occupational exposure to HIV (Suglo et al. 2021:210). Midwifery practitioners are among the HCWs with high statistical association with risk for exposure to HIV (Tsega et al. 2023:7). However, the report from OHS practitioners varies vastly from the account from midwifery practitioners, as quoted further in the text:

‘… incidences happen in the labour unit, for example, not so long ago, I was suturing the woman’s perineum and realised that there was blood inside the glove.’ (Participant 7, midwifery practitioner, H1)

‘… the most common exposure in labour ward is through needle pricks during suturing.’ (Participant 6, midwifery practitioner, H3)

‘I once had liquor splash on my face, but because I was wearing a mask and my glasses, I just washed off the face.’ (Participant 3, midwifery practitioner, H3)

##### Knowledge of post-exposure prophylaxis benefits and guidelines

The participants displayed some knowledge of the PEP guidelines even though implementation seemed to be a challenge. The participants were aware of the effectiveness of PEP and agreed that PEP could potentially prolong life. This is congruent with the study in Gondor, Ethiopia, where 98.5% of participants agreed on the importance of PEP for HIV, and 78.5% believed it can reduce the probability of being infected (Mmeremikwu et al. 2020:37):

‘It is very important that one completes the course despite the side effects of the ARVs to fully prevent HIV infection.’ (Participant 2, midwifery practitioner, H2)

##### Knowledge of what needs to be done immediately after exposure

First aid provides the participants with reassurance that the likelihood of transmission is reduced. The participants reported that their immediate response to the exposure is to perform first aid, which includes putting the affected finger under running tap water. This is congruent with the findings of Suglo et al. (2021:210), where participants who had experienced either a needle-stick injury or a blood splash during work would wash the site of injury with soap and under running water. At the same time, other midwifery practitioners squeezed the blood out of the cut, while others did not:

‘Immediately after I was pricked while suturing the woman, I went to the tap and ran the water over the cut while squeezing the blood out.’ (Participant 2, midwifery practitioner, H2)

#### Theme 2: Reporting incidents and seeking treatment

While all participants were aware of the importance of reporting accidental exposures, non-compliance was noted from some participants.

##### Motivation to report the incident

Accidental exposure to BBFs is regarded as an injury that must be reported as per the *Compensation for Occupational Injuries and Disease Act* (*COIDA*). According to the OHS practitioners, the requirement to complete injury on duty (IOD) forms as part of the procedure could be one of the main reasons why midwifery practitioners report incidents. Some are genuinely scared for their health and would report that they can start a PEP regimen as soon as possible:

‘I usually get to know about the exposed individual from the registry department where they had opened a file and completed injury on duty forms in accordance with the *COIDA*.’ (Participant 5, OHS practitioner, H2)

‘The only circumstance I would see a midwife is when they bring the injury on duty forms because I have to sign and facilitate the submission in case of compensation.’ (Participant 6, midwifery practitioner, H3)

##### Reluctance to report the incident

The procedure to report includes starting at the accident and emergency department, where a clinician will procedurally do counselling, draw blood from the affected individual and determine the source patient’s HIV status, wait for the blood results and if tested negative, be provided with PEP therapy. The reporting continues to the OHS unit to complete the IOD forms in accordance with the *COIDA*. The participants are aware and understand the importance of following guidelines but are just reluctant to report because of the tedious process, unlike in the study among HCWs in Pakistan, where the major reasons for not reporting were a lack of knowledge of policies for reporting, fear of stigma and discrimination, a lack of support and motivation (Shakeel et al. 2022:5):

‘You know the midwives don’t like to report, I was also a midwife before working here, but I believe they will come because they need me to complete their injury on duty forms. In the five years I’ve been working here, I haven’t really had a midwife coming to me for this. I believe there are no incidences to report, or they just don’t report.’ (Participant 6, midwifery practitioner, H3)

##### Barriers to seeking treatment at the facility

The waiting times at the accident and emergency department discourage those who need to see the doctor for side effect management. Even though priority will be given to staff members, the waiting is still considered too long:

‘I would rather use my own medical aid to see my doctor than come to work and wait at casualty to be seen after a long wait.’ (Participant 13, midwifery practitioner, H2)

‘Management of side effects was achieved through consulting with my private doctor. A colleague advised that going to casualty would cause me to wait longer, which I experienced when I went there for the first time to report the incident and test for HIV.’ (Participant 9, midwifery practitioner, H1)

#### Theme 3: Support

The anxiety that may result from the thought of a potential infection with HIV after direct exposure to BBFs requires good support. The exposed individuals could go through various emotions, including a fear of following the process, which requires them to also check their status before administering PEP.

##### Role of occupational health and safety practitioners

Occupational health and safety practitioners are responsible for providing health and safety programmes and services to workers and focus on promoting and preventing illness and injury. Moreover, they protect against risks related to work and the environment. It seems that OHS has not been given much attention in the developing countries (Tawiah et al. 2022:2). The OHS practitioners have a broader role in the selected hospitals. They are involved in other safety domains, such as infrastructure safety, and participation in committees like Quality, infection prevention and control (IPC) and more. The responsibility lies on the OHS team to arrange training for all employees to provide them with knowledge on health and safety matters (Denge & Rakhudu 2022:7). The immediate reporting of the exposures to BBFs is channelled to the accident and emergency department because of their 24-h service delivery and presence of a clinician to carry out the immediate management and prescription of antiretroviral therapy (ART):

‘Part of my duty is to coordinate and facilitate the reporting of injury on duty and help the personnel member to complete the relevant forms.’ (Participant 4, OHS practitioner, H1)

‘After identifying personnel who reported to casualty after injury, I follow up and remind them to come back to report any side effects and do follow-up tests.’ (Participant 5, OHS practitioner, H1)

##### Importance of peer support

In a community where people work together, it is easy to influence one another’s behaviour to provide support. Midwifery practitioners spend much of their time at work because of long hours per shift. They tend to rely on one another for support. When these exposures occur, they report to one another before they decide to report. The colleagues assess the level of danger and influence the exposed individual on what needs to be done:

‘We guide each other about what to do post exposure, starting with performing first aid and then reporting.’ (Participant 6, midwifery practitioner, H3)

‘… when I was taking my PEP, I suffered side effects, the worst was insomnia, and my colleagues advised me to change the times for taking the meds, and it got better.’ (Participant 7, midwifery practitioner, H1)

##### Lack of staff for counselling

In the selected sites of the study, counselling is provided by the casualty clinician before drawing blood from the exposed individual. However, the participants feel that counselling is not optimal to alleviate the anxieties related to potential seroconversion. It is crucial to provide adherence counselling to ensure maximisation of adherence to PEP because of universal evidence that most exposed individuals are reported to be afraid of the side effects (Tarimo & Mashoto 2019:102):

‘Counselling is never proper. The doctor at casualty is always busy and does not only see the staff but patients too. You get to casualty and wait for the doctor, and when he comes, he talks while taking blood, no time to allay the anxieties you might be having.’ (Participant 9, midwifery practitioner, H1)

#### Integration of quantitative and qualitative results

This mixed-methods study followed a nested or embedded approach, where the qualitative data were sought to augment the outcomes of the study, which is a popular approach within implementation and dissemination research (Agency for Healthcare Research and Quality [AHRQ] 2013:2). Quantitative and qualitative data were collected concurrently; however, one was a main method to guide the project and the other a secondary database (Santos et al. 2017:4). Using a convergent design and a merging approach to integration, the researcher presented results with a themes-by-statistics display to array themes from the participants’ experience of and their perceptions about HIV PEP. The convergent design involves quantitative and qualitative data collection and analysis at similar times, followed by an integrated analysis (Guetterman, Fetters & Cresswell 2015:555). The synopsis of integrated results is discussed in Table 3 under the following: Knowledge about PEP, attitudes and perceptions about PEP and compliance with PEP guidelines.

**TABLE 3:** Quantitative and qualitative integrated results.

Integrated results from quantitative and qualitative strands
**Knowledge about PEP** There were similarities between the quantitative and qualitative data regarding the responses related to knowledge of PEP guidelines. All the participants displayed good knowledge of HIV PEP as all questions asked to determine their degree of knowledge were answered positively. What was found was that the guidelines were not completely complied with. Another congruency in this mixed-methods data was that there was a suboptimal level of reporting, and this was confirmed by the OHS practitioner, who indicated that midwifery practitioners do not like to report, and the midwives themselves, who stated that they do not report regularly.
**Attitudes and perceptions about PEP** Attitudes of participants and respondents towards the use of PEP were positive from the quantitative and qualitative strands. About 99% of respondents agreed to reporting the accidental exposures to BBF’s and the use of PEP if indicated. They also believed that PEP should be started as soon as possible. This was corroborated by the face-to-face interview participant, who indicated the importance of reporting and starting with PEP immediately after the BBF exposure and making sure that the course is completed despite experiencing side effects.
**Compliance with PEP guidelines** Evidently, in both the qualitative and quantitative data collected, there was no full compliance with PEP guidelines. The completion of the PEP course was accounted for by 66% of the 74% who started the treatment. This was supported by a midwifery practitioner, who mentioned that side effects were the reason she decided to stop the therapy. She further elaborated that after experiencing nightmares and remembering the mental effect of PEP as discussed with colleagues, she then stopped taking them before she could experience hallucinations.

*Source:* Matlala, M.S., 2023, ‘Strategies to improve compliance to post exposure prophylaxis guidelines for midwifery practitioners at specific hospitals in Gauteng province, South Africa’, PhD thesis, Dept. of Health Studies, University of South Africa.

BBF, blood and body fluids; PEP, post-exposure prophylaxis; HIV, human immunodeficiency virus; OHS, occupational health and safety.

## Discussion

Exposure to BBFs is inherent in midwifery practice, especially in the labour ward, where, during delivery, amniotic fluid and blood are certainly featured. It is, therefore, crucial that all HCWs in risky environments take the necessary precautions. It is a concern that some midwifery practitioners may not report the incidents of exposure, which means they will not get the prophylactic treatment for HIV.

This mixed-methods study revealed that the midwifery practitioners have sufficient knowledge about PEP but still lack commitment to full compliance. This is congruent with the findings in Eastern Ethiopia, where most HCWs were found to have good knowledge of PEP (Eticha & Gemeda 2019:2). Studies conducted in Southern Africa have demonstrated that HCWs had knowledge about PEP; nevertheless, simply hearing about PEP does not provide a reliable indicator of one’s understanding of PEP measures and treatment (Makhado et al. 2022:5). This is unlike findings among nurses in Bhutan, where only 51.1% had heard about PEP against HIV.

A national study in Kenya also showed, among those who were knowledgeable, only 45% sought HIV PEP, indicating that the main reasons for not seeking PEP among this group were a lack of sufficient information, fear of the process, and what could follow (Bareki & Tenego 2018:2). A gap in reporting and attaining PEP was also reported in one South African study among nurses (Rasweswe & Peu 2020:4).

### Prevalence of occupational exposure to blood and body fluids in maternity units

Incidents of occupational exposure to BBFs are high in the maternity units. Over 70% of participants in this study were directly exposed to BBFs, which is much higher than what was reported in Butajira Town, Southern Ethiopia, where 7.9% of the cohort practising midwifery was exposed (Mulatu & Hussen 2019:3). This outcome was also higher than in Tanzania where 50.6% participants experienced occupational exposure (Kimaro et al. 2018:1).

The procedure of reporting accidental exposures is widely known in the maternity units of the selected sites. This is, unlike in Ghana, where 10% of participants’ reasons for not reporting included participants not knowing whom to report to (Suglo et al. 2021:210). This study found that among the 71% of participants who were directly exposed to BBFs, 70% reported the incidents, which is higher than what was reported in Tanzania’s Singida Region, of 121 exposed participants, 83 reported the exposure incidents to management (Kimaro et al. 2018:5).

Placing an exposed area under running tap water while squeezing was common practice among participants. However, squeezing is not recommended, as it may cause further tissue injury, which may increase the risk of infection (Babanawo et al. 2018:8).

The current study detected high levels of occupational exposures with less reporting behaviour. Reporting BBF’s exposures has many values, such as the fact that it allows the affected HCW to receive appropriate and prompt medical assessment, counselling and treatment and PEP and enables the award of appropriate compensation as prescribed in the *COIDA* (Mbah, Elabor & Omole 2020:3). Low reporting rates provide wrong statistics and underreported exposures will remain unknown, leading to low rates of PEP uptake (Rasweswe & Peu 2020:4).

### Perceptions about post-exposure prophylaxis

A larger proportion (86%) of participants had a positive attitude towards PEP, meaning they would opt to use PEP should they experience direct occupational exposure. Even though some participants in the qualitative strand did not report their exposure, it was not that they had a negative attitude towards PEP. It was either that they took advantage of the fact that the source was negative or that they sought services elsewhere. Although a positive attitude towards PEP is commendable, it can only be appreciated when it translates to good knowledge and practice (Mmeremikwu et al. 2020:37). A positive attitude towards PEP was expressed with the emphasis that it is a short course and is effective in reducing the likelihood of contracting HIV. This finding is aligned with the findings of South-Eastern Nigerian HCWs (96.2%), giving affirmation of the positive effect of PEP to reduce HIV infection (Mmeremikwu et al. 2020:37).

### Compliance with human immunodeficiency virus post-exposure prophylaxis

First aid, counselling, risk assessment, HIV testing, obtaining the exposed person’s informed consent and maintaining confidentiality are all included in PEP (Makhado & Seakane 2020:9). This study confirmed that midwifery practitioners do not always comply with the PEP guidelines, which the OHS practitioners in this study supported. They reported that only a few midwifery practitioners presented at their units to report accidental exposure.

While a significant number of midwifery practitioners are accidentally exposed to BBFs during their routine duties, approximately 72% completed the course of therapy, some citing side effects as the reason for non-completion. This is higher as compared with the findings in Tanzania where more than half of the respondents had taken PEP had completed the course (Kimaro et al. 2018:5). In Ethiopia, among the 61.6% who had experienced occupational exposure, only 24.3% had used HIV PEP (Degavi et al. 2020:4).

Literature shows that studies conducted in many countries reveal a low uptake of PEP among HCWs. The findings of this study are slightly higher than the CDC’s estimation that 17% – 47% of health professionals who take PEP after occupational exposure did not complete a full 4-week course of therapy because of an inability to tolerate the drugs (Mulatu & Hussen 2019:4).

Although it is appreciated that some participants sought PEP services elsewhere, it leaves a gap in the overall capturing of reported cases. Reporting exposures will also provide facilities with accurate data and the rate of seroconversion, which could be used for planning in-service training, workshops and seminars on HIV PEP (Babanawo et al. 2018:8).

The World Health Organization (WHO) recommends that all healthcare institutions should have a 24-h, easily accessible system that enables reporting and managing HCWs who experience occupational exposure to HIV (Bareki & Tenego 2018:2). Most participants in this study admitted to having good access to 24-h PEP services on site, which is unlike in other places such as Bhutan’s tertiary hospital, where poor PEP services in the hospital and a lack of support to report exposures were reported to be the two leading causes resulting in low uptake (Tshering, Wangchuk & Letho 2020:7).

This study found that although participants were aware of PEP guidelines and the availability of PEP onsite, there was a huge gap in reporting, implementation and practice of the guidelines. This is consistent with the findings of Rasweswe and Peu (2020:4), among Gauteng nurses on using PEP, where a significant gap was identified between HIV PEP guidelines and the implementation and practice thereof.

### Limitations

This study was conducted among midwifery practitioners in only three hospitals in the same district in the Gauteng province. Therefore, the findings may not represent all midwifery practitioners in the country. However, the findings painted a clearer picture of the PEP knowledge and the behaviour among midwifery practitioners, which is likely to be the same in other settings, as supported by many studies conducted among HCWs. Another limitation is that three OHS practitioners were sampled to represent each study site, but only two participated, which means only two hospitals were represented.

### Recommendations

Several studies conducted among nurses and midwifery practitioners globally yielded results similar to this study. The challenges of underreporting incidents of exposure to BBFs, underutilisation of PEP services and a lack of training on PEP guidelines pose a threat to the quality of health for the frontliners, who are also the backbone of the health system. It is therefore recommended that:

- The South African Department of Health should prioritise the implementation of critical programmes related to safeguarding the well-being of clinical cadres, including the mandatory training package about the PEP guidelines and all other essential guidelines.
- Application of strategies and other models of compliance to inform policies and protocols for the management and mitigation of safety risks at the clinical workplace should be made.
- A further prospective cohort study should be conducted in other provinces of SA to derive a generalisation of common findings.

## Conclusion

The prevalence of accidental exposures to BBFs in maternity units is high and requires effective management through proper interventions. There is a need for interventions to reduce the burden of healthcare-related infections and to set up effective surveillance programmes to determine their impact (Bekele, Yimam & Akele 2018:1). Training remains the most effective method to inculcate the culture of learning and practice. Underutilisation of the PEP services indicates either a gap in knowledge, a lack of trust in the effectiveness of PEP, self-testing or even negligence on the side of the midwifery practitioners. Protecting HCWs requires institutionalisation of occupational health risk assessment and risk-based medical surveillance (Mossburg et al. 2019:10). Long waiting times prior to receiving help discourage newly exposed midwifery practitioners in that they either seek help elsewhere or perhaps do not even follow through with the process. The provision of PEP services needs to be reviewed to achieve universal coverage for HCWs at risk of HIV infection through accidental exposures to BBFs. Providing PEP alone to these exposed people is not sufficient due to initiation time, type of recommended regimen, completeness and follow-up are determinant factors and crucial for PEP effectiveness (Temesgen, Weldu, Getahun & Aragaw 2020:69).
